# Effect of Pain Reduction and Functional Improvements Following a Noninvasive Biomechanical Intervention for Gait Rehabilitation on Healthcare Claims: An Observational Study

**DOI:** 10.36469/001c.140740

**Published:** 2025-06-19

**Authors:** William Vanderveer, Eric Freeman

**Affiliations:** 1 Redefine Healthcare, Orthopedic Pain & Spine Center, Matawan, New Jersey, USA

**Keywords:** knee pain, low back pain, biomechanics, claims analysis, gait rehabilitation

## Abstract

**Background:** Musculoskeletal conditions substantially impact public health in the United States, affecting approximately 128 million adults and resulting in over $600 billion in annual socioeconomic costs. Low back pain and knee osteoarthritis are the most prevalent musculoskeletal disorders, with projections suggesting their incidence will increase markedly due to aging populations and rising obesity rates.

**Objective:** The purpose of this study was to evaluate healthcare claims utilization (HCRU), clinical outcomes, and patient satisfaction for individuals with knee and back pain treated with a home-based, biomechanical intervention that aims to provide precision medicine for patients with musculoskeletal conditions.

**Methods:** A retrospective analysis of claims data was conducted on 616 patients with knee and back pain who were treated with a noninvasive, home-based, biomechanical intervention (AposHealth) from October 2020 to October 2023. Eligibility was determined based on specific diagnostic criteria. The controls were 3576 patients with knee and back pain who were receiving the standard of care. HCRU, pain levels, functional disability, and patient satisfaction were captured.

**Results:** Significant reductions in HCRU were noted, with significantly lower rates of minor and major surgeries compared with control groups. The economic analysis suggested substantial cost savings of approximately $9 million over 20 months, suggesting an 80% reduction in costs in those treated with the biomechanical intervention compared with controls. Pain levels decreased significantly by 32.5% and 57% at 3 and 6 months posttreatment for back pain, respectively. Patients with knee pain reported a significant decrease of 39% and 35% at 3 and 6 months, respectively.

**Discussion:** The examined biomechanical intervention led to a significant reduction in pain and improvement in function, which presumably is a driving factor for a meaningful reduction in HCRU and potential cost savings.

**Conclusions:** There is an urgent need for innovative strategies that alleviate the burden of musculoskeletal disorders on the healthcare system. The results of this study add to the evidence about the clinical effectiveness and cost-savings of this intervention in patients with knee and back pain, using real-world data.

## INTRODUCTION

Musculoskeletal conditions represent a significant public health issue in the United States, affecting millions of individuals and leading to substantial socioeconomic burden.^1^ Epidemiological studies indicate that approximately 128 million adults in the United States suffer from chronic back and joint pain.^2^ The economic burden of these conditions is staggering, with estimates suggesting that they account for roughly $400 billion in annual healthcare costs, lost productivity, and other related expenses.^3^ Accounting for the rapidly aging population worldwide due to declining birth rates and rising life expectancies, proactively addressing the array of public health challenges is essential.^4^

Among the various musculoskeletal conditions, low back pain (LBP) and knee osteoarthritis (OA) are particularly prominent, posing significant challenges both to individuals and the healthcare system.^2^ LBP affects nearly 80% of adults at some point in their lives, making it one of the most prevalent health complaints,^5^ with over 10% of them suffering from chronic back pain.^6^ Similarly, knee OA is a common degenerative condition that affects 20% of American adults aged 45 years and older,^7^ approximately 26 million US adults, leading to increased pain, decreased mobility, and diminished quality of life. Projections indicate that as the population ages and obesity rates continue to rise, the prevalence of LBP and knee OA will likely increase by 30% or more over the next 2 decades.^1^ This surge in numbers is expected to amplify the economic burden associated with these conditions, potentially exceeding $1 trillion annually by 2030 in the United States alone,^1^ considering direct medical costs and indirect costs related to lost productivity. The main driver of healthcare cost expenditure is surgery.^8^ The implications for society are profound, as increasing rates of disability and the associated healthcare demands could overwhelm healthcare resources, necessitating a shift toward innovative and cost-effective approaches for prevention and management.

Musculoskeletal biomechanics involves understanding how the forces exerted on muscles, bones, and joints contribute to the development and progression of these disorders. Abnormal biomechanics, such as altered joint alignment, abnormal neuromuscular control, and movement patterns, can lead to increased stress on specific structures, ultimately resulting in pain, inflammation, functional limitations and ongoing deterioration of degenerative processes.^9^ Current biomechanical interventions aim to address these abnormalities, often focusing on optimizing joint loading, improving movement efficiency, and enhancing stability. Examples of such interventions include orthotic devices, insoles, physical therapy techniques, and surgical options like realignment procedures. While many of these approaches have shown varying degrees of effectiveness, mostly minor and short-lived symptomatic relief, there remains a need for noninvasive solutions that provide lasting benefits without the risks associated with more invasive procedures.^9–11^

A home-based foot-worn biomechanical device (AposHealth) emerges as a promising noninvasive biomechanical intervention developed to address musculoskeletal conditions, primarily LBP and knee OA,^12,13^ by personalizing the treatment to address each patient’s specific needs. The program utilizes adjustable footwear designed to promote proper alignment^14–17^ and train neuromuscular control^18–20^ to optimize biomechanics during physical activity. An existing randomized controlled trial (RCT) has demonstrated the efficacy of AposHealth for knee OA, revealing significant improvements in pain levels and functional outcomes compared with standard care.^12^ Encouragingly, a new RCT was recently published demonstrating a superior effect of Apos compared with traditional physical therapy in patients with chronic LBP.^13^ One retrospective study looked at changes in claims before and after treatment with Apos in members covered by a large payer in New York City and demonstrated a reduction in overall healthcare resource utilization.^21^ Other studies reported decreased rates of total knee replacements.^22–26^

The purpose of this study was to look at the clinical outcomes, healthcare claims utilization (HCRU), and patient satisfaction in patients with knee and back pain treated in a single clinical practice. By examining both clinical and economic outcomes, we seek to contribute additional evidence on economic data of a large payer in New Jersey, regarding the benefits of this intervention as a viable treatment option for individuals with chronic musculoskeletal conditions.

## METHODS

This retrospective analysis examined claims data of individuals covered by a state-based plan who underwent treatment for knee and/or back pain. The treatment utilizes a noninvasive, home-based biomechanical solution (AposHealth®) for gait rehabilitation and training neuromuscular control. Eligibility for participation was based on specific diagnostic criteria. The intervention was deemed medically necessary if patients exhibited moderate to severe knee joint disease (ie, knee OA or persistent LBP symptoms). A detailed outline of the inclusion criteria can be found in **Table 1**, as previously described by Mark et al.^21^ The clinical service and data for this analysis were provided and obtained from a provider specializing in orthopedics, spine, and pain management located in New Jersey.

**Table 1.** Eligibility Criteria of the Home-Based, Noninvasive Biomechanical Intervention

**Medically Necessary**	**Not Medically Necessary**
A diagnosis of moderate or advanced knee joint disease or low back pain supported by a clinical diagnosis Specifically, patients with the following diagnostic codes were included: M170, M1710, M1711, M1712, M179, M25561, M25562, M25569, M47817, M5136, M5186, M5416, M5417, M5430, M5431, M5432 M5441, M5451, M549 Corresponding to the following indications: bilateral primary osteoarthritis of knee; unilateral primary osteoarthritis, unspecified knee; unilateral primary osteoarthritis, right knee; unilateral primary osteoarthritis, left knee; osteoarthritis of knee, unspecified; pain in right knee; pain in left knee; pain in unspecified knee; spondylosis without myelopathy or radiculopathy, lumbosacral region; other intervertebral disc degeneration, lumbar region; other intervertebral disc disorders, lumbar region; radiculopathy, lumbar region; radiculopathy, lumbosacral region; sciatica, unspecified side; sciatica, right side; sciatica, left side; lumbago with sciatica, right side; lumbago with sciatica, left side; vertebrogenic low back pain; dorsalgia, unspecified Documentation of persistent pain that is not controlled despite optimal, conservative pain management: To include a description of the pain (onset, character, aggravating, duration, and relief factors), analgesics and the treatment modalities used; and Documentation of functional limitations that interfere with ADLs: To include the specific limitation of ADLs; To include an evaluation of safety issues, eg, fall risk; and Documentation of a history of conservative medical therapy that has been tried and failed including but not limited to ≥1 of the following: Activity modification; or Structured land-based programs including strengthening and/or cardio and/or balance training/neuromuscular exercise; or Mind-body exercise including tai chi or yoga; or Physical therapy that includes flexibility and muscle strengthening exercises; or NSAIDs; or Therapeutic intra-articular injections as appropriate; or Weight loss efforts as appropriate	The interventions will be considered contra-indicated and not medically necessary with the presence of the following: Active infection of the knee joint or back or an active infectious process anywhere in the body (eg, systemic bacteremia); or Patient requires a cane or walker both indoors and outdoors Patient has a history of ≥2 unexplained falls within the last 12 months Patient has severe neurological, psychiatric, or comprehension issues preventing an understanding of how to use the device Patient has severe balance or vertigo issues

Participants received treatment between October 2020 and October 2023. Potential candidates were identified within the clinics. If individuals were diagnosed with a primary knee or back condition and met eligibility requirements, a physical therapist would initiate a clinical assessment and configure the device according to established methodology and protocols, as elaborated below. All participants provided consent, acknowledging that their information could be utilized anonymously for research purposes, and were assigned a de-identified code for data analysis to ensure anonymity.

To serve as a comparison, we included members with similar profiles who were treated by the same provider but did not receive the biomechanical interventions. The control group received the current standard of care for patients with knee and back pain.

### Outcomes and Data Collection

HCRU was determined by monitoring claims related to diagnoses or procedures for knee OA and LBP, categorized into the following groups:

1. Total knee replacement
2. Major back surgery
3. Minor knee surgery
4. Minor back surgery
5. Knee/back injections
6. Physical therapy

A detailed summary of claims codes is provided in **Table 2**.

**Table 2.** Claims Codes

**Category**	**Procedure**	**Codes**
Back injections	Corticosteroid - general	J0702, J1020, J1030, J1040, J1094, J1100, J1700, J1710, J1720, J2650, J2930, J3300, J3301, J3302, J3303, J3304
	Hyaluronic acid	J3470, J3471, J3472, J3473, J7316, J7318, J7320, J7321, J7322, J7323, J7324, J7325, J7326, J7327, J7328, J7329
	Injection - general large joint	20552, 20553, 27096, 62282, 62323, 62327, 64483, 64484, 64493, 64494, 64495
	PRP	0232T
Back nonoperative treatment	Back orthosis	L0452, L0454, L0455, L0456, L0457, L0458, L0460, L0462, L0464, L1005, L1010, L1020, L1025, L1030, L1040, L1050, L1200, L1300, L1310
	Physical therapy	97161, 97162, 97163, 97164
Back surgery	Disc replacement	22857, 22862, 0163T, 0164T, 0165T
	Discectomy	22224, 62380, S2350, S2351
	Fusion	22533, 22534, 22558, 22585, 22586, 22612, 22630, 22633
	Laminectomy	22102, 63005, 63012, 63017, 63030, 63035, 63042, 63044, 63047, 63056, 63200, 63252, 63267, 63268, 63272, 63273, 63277, 63278, 63282, 63283, 63287, 0202T, 0275T
	Osteotomy	22214, 22224
Knee injections	Corticosteroid - general	J0702, J1020, J1030, J1040, J1094, J1095, J1100, J1700, J1710, J1720, J2650, J2920, J2930, J3300, J3301, J3302, J3303, J3304
	Hyaluronic acid	J3470, J3471, J3472, J3473, J7318, J7320, J7321, J7322, J7323, J7324, J7325, J7326, J7327, J7328, J7329
	Injection - general large joint	20552, 20553, 20610, 20611
	PRP	0232T
Knee nonoperative treatment	Knee orthosis	E0935, L1810, L1812, L1820, L1830, L1831, L1832, L1833, L1834, L1836, L1840, L1843, L1844, L1845, L1846, L1847, L1848, L1850, L1851, L1852
	Physical therapy	97161, 97162, 97163, 97164
Knee surgery	Removal/revision knee arthroplasty	20680, 24787, 27486
	Total knee replacement	27445, 27446, 27447
Therapeutic arthroscopy and other minor back surgery	Discography - myelography	62304, 62305, 72265, 72295
	Other	64625, 64635, 64636
Therapeutic arthroscopy and other minor knee surgery	Arthroscopy	29866, 29867, 29868, 29871, 29873, 29874, 29875, 29876, 29877, 29879, 29880, 29881, 29882, 29883, 29884, 29885, 29886, 29887, G0289
	Arthrotomy	27331, 27332, 27333, 27334, 27335, 27403, 27407
	Other	27310, 27347, 27415, 27416, 27570, G0428

Additionally, modifications in pain levels and functional disability were evaluated at baseline (before treatment commencement) and after 6 months using a visual analog scale pain scale between 0 mm and 100 mm (or 0 cm–10 cm) for all patients and the Oswestry Disability Index for patients with primary back pain. Both tools have been recommended for use in clinical research and practice and contain high reliability and validity.^27–29^ In addition, gait velocity was assessed using a mobile-based digital gait analysis^30^ to provide an objective evaluation of the patient’s functional condition. Patient satisfaction was measured at the end of the program using the question, “How satisfied were you with the Apos treatment?” Patients were asked to rate their level of satisfaction on a scale between 0 and 10, where 0 = not satisfied at all and 10 = very satisfied. Patients were instructed to complete questionnaires when they arrived at the clinic for their initial evaluation and when returning to follow-up appointments at 3 and 6 months. The physical therapist provided instructions and support to patients as required. The physical therapist also conducted a computerized gait test using an artificial intelligence–driven smartphone-based gait analysis application (Celloscope Ltd., Tel Aviv, Israel) on all patients at initial evaluation and follow-up appointments. Patients were asked to walk barefoot, at a self-selected pace, along a 10-meter walkway in 2 consecutive trials. Gait velocity (cm/s) was calculated for every gait test and served for further analysis.

### Intervention

**Figure 1.**
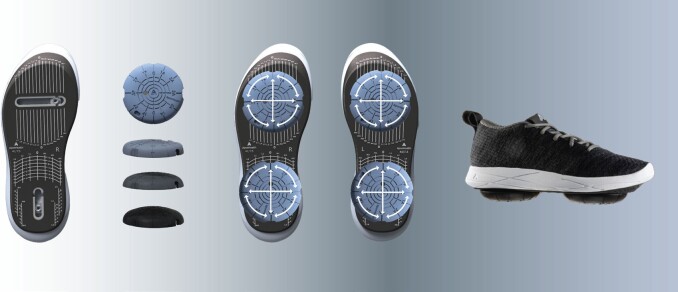
Biomechanical Device

Each participant received an FDA-cleared, personalized, calibrated foot-worn device (Apos®) and a home-based treatment program designed to relieve pain and enhance function for individuals with musculoskeletal issues. The treatment comprised a unique footwear device (Apos® device; see **Figure 1**) featuring 2 convex pods fixed to the bottom of the sole. A qualified clinician customized the device by adjusting the pod locations and sizes, accommodating for gait abnormalities, compensations, and clinical symptoms to alleviate knee or back pain and retrain neuromuscular control. The calibration of the device was individually tailored to achieve functional realignment, redistributing loads across the kinematic chain while stimulating neuromuscular activity to retrain muscle control.

Shifting the pod locations adjusts the foot’s center of pressure, redirects the ground reaction force vector, and provides immediate pressure relief on the joints.^14,15,17,31^ The convex design of the pods introduces controlled instability by narrowing the foot’s base of support, challenging gait and postural stability.^18–20^ This intentional instability activates a neuromuscular response, prompting changes in patients’ walking patterns.^14,17^ After calibration, patients were advised to wear the device daily for progressively longer periods during specific activities (eg, daily tasks) at home or work,initially for about 20 minutes, gradually increasing to 1 to 2 hours daily. This home-based/work-based training empowers patients to manage their symptoms while incorporating the exercise into their regular routines, promoting a beneficial effect even while walking without the device over time. Patients were also encouraged to attend follow-up appointments for further assessment of clinical outcomes and gait patterns, allowing for adjustments to the device calibration and treatment plan as necessary. Generally, patients were instructed to return for 2 to 3 follow-up visits, either virtually or on-site.

### Statistical Analysis

Descriptive statistics, including frequencies, means, and SD, to present patient characteristics, alongside a count of HCRU claims, were employed. A comparison between the intervention and control groups was performed. The ANOVA tests were used to compare the number of claims between the 2 groups. The difference between the groups for each pain area separately was further examined with the chi-square tests. To take into account the entire sample that includes subjects with missing data we used the mixed models for repeated measures, with diagonal covariance structure and index of 2 time points. We used the linear mixed model to present improvements over time in velocity, pain and Oswestry Index. We selected variables that define the subjects, repeated observations over time and the covariance structure for the residuals. Data were analyzed with IBM SPSS statistics software version 29.0. (SPSS Inc.). Significance levels were set at .05.

## RESULTS

With respect to claims data, a total of 616 patients received the intervention. Of them, 383 (62%) patients had primary back pain; 155 (25%) patients had primary knee pain; and 78 (13%) patients had a combination of knee-spine pain. Claim analysis was done on average (SD) after 606 (285) days of treatment. Patients’ mean (SD) age was 53.6 (11.4) years, and the mean (SD) body mass index was 32.5 (7.9). Regarding gender, 67% of the patients were female, 32% were male, and 1% were other. More specifically, for patients with back, knee, and knee-spine pain, the claims analysis had an average (SD) of 557 (280) days, 470 (262) days, and 755 (243) days, respectively. A total of 3576 patients served as controls. Of them, 2938 (82%) patients had primary back pain. 430 (12%) patients had primary knee pain, and 208 (6%) patients had a combination of knee-spine pain. With respect to clinical outcomes at baseline, some data were missing. Results are summarized in **Table 3**. Data on treatment adherence were available for 153 patients and suggested that most patients (73%) used the device 4 to 7 days a week, 13% use the device 1 to 3 days a week, and the rest (14%) used the device less than once a week or not at all.

**Table 3.** Clinical Outcomes and Gait Velocity at Baseline (Treatment Initiation)

	**Back**	**Knee**
Pain (score 0-100)	41.2 (24.8)	42.9 (24.3)
Oswestry Index (score 0-100)	37.5 (16.5)	–
Gait velocity (cm/s)	87.4 (19.3)	84.5 (19.1)

Results are presented as mean (SD).

**Figure 2.**
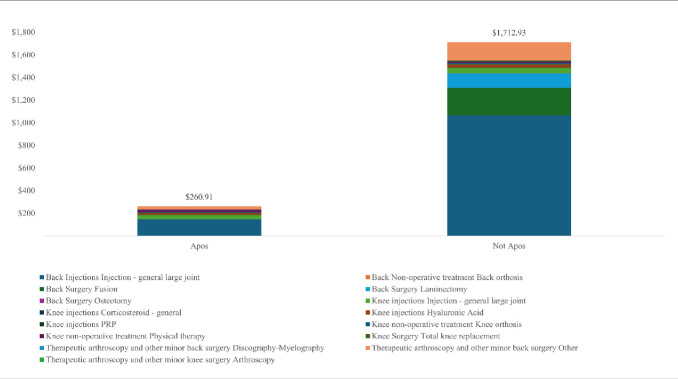
Total Collections per Patient

A significant reduction was seen in HCRU after the utilization of the new intervention in all groups. Overall, none of the patients had major or minor knee surgery in those treated with the device compared with 0.4% and 0.3% for major and minor knee surgery, respectively, in the control group. Of the patients treated with the biomechanical device, 0.2% and 1.6% had major or minor back surgery, respectively, compared with 4.4% and 8.1% of major and minor back surgery, respectively, in controls. Of the patients treated with the biomechanical interventions, 18.8% had claims of knee/back injections compared with 81.7% in the control group. Of the patients treated with the biomechanical treatment, 10.7% had physical therapy compared with 18.1% of controls. A subgroup analysis of primary knee pain, primary back pain, and knee-spine pain is summarized in **Table 4**. In addition, **Figure 2** illustrates the total collections per patient and breakdown by categories in patients treated with the biomechanical device and controls.

**Table 4.** Count of Claims Data for Patients With Primary Knee Pain, Primary Back Pain, and Knee-Spine Pain

	**Biomechanical Treatment**					**Controls**				**P Value**		
	**Total**	**Back**	**Knee**	**Knee-Spine**	**Total**	**Back**	**Knee**	**Knee-Spine**	**Total**	**Back**	**Knee**	**Knee-Spine**
N	616	383	155	78	3,76	2938	430	208				
Total knee replacement, n (%)	0 (0.0)	0 (0.0)	0 (0.0)	0 (0.0)	15 (0.4)	0 (0.0)	14 (3.3)	1 (0.5)	.244			.99
Major back surgery, n (%)	1 (0.001)	1 (0.3)	0 (0.0)	0 (0.0)	157 (4.4)	153 (5.2)	0 (0.0)	4 (1.9)	<.001	<.001		.578
Minor knee surgery, n (%)	0 (0.0)	0 (0.0)	0 (0.0)	0 (0.0)	11 (0.3)	0 (0.0)	11 (2.6)	0 (0.0)	.339		.043	
Minor back surgery, n (%)	10 (1.6)	6 (1.6)	0 (0.0)	4 (5.1)	290 (8.1)	270 (9.2)	0 (0.0)	20 (9.6)	<.001	<.001		.337
Knee/back injections, n (%)	40 (6.5)	8 (21.9)	7 (4.5)	25 (32.1)	2922 (81.7)	2446 (83.3)	299 (69.5)	17 (85.1)	<.001	<.001	<.001	<.001
Physical therapy, n (%)	66 (10.7)	40 (10.4)	7 (4.5)	19 (24.4)	648 (18.1)	515 (17.5)	80 (18.6)	53 (25.5)	<.001	<.001	<.001	.88

Subgroup includes those with primary back pain, primary knee pain, and knee-spine pain.

The linear mixed model suggested significant improvement over time in pain (*P*<.001), Oswestry Index score (*P* = .009), and gait velocity (*P* < .001). For patients with back pain, pain decreased by 32.5% and 57% at 3- and 6-month posttreatment initiation, respectively. For patients with knee pain, the improvement was 39% and 35% for 3- and 6-month posttreatment initiation, respectively. Oswestry Index improved by 17% and 32.8% for 3- and 6-month posttreatment initiation, respectively, in those with primary back pain. For patients with back pain, gait velocity has increased by 6.5% and 13.6% at 3- and 6-month posttreatment initiation, respectively. For patients with knee pain, the improvement was 5.2% and 7.5% for 3- and 6-month posttreatment initiation. With respect to patient satisfaction, the average (SD) satisfaction was 8.3 (2.2) of 10 in 112 patients who answered this question.

## DISCUSSION

This study demonstrated that utilizing a noninvasive, home-based/work-based biomechanical device and treatment resulted in a significant reduction in HCRU in patients with chronic knee and back pain. Given the pressing need for effective interventions aimed at mitigating the prevalence and impact of musculoskeletal disorders, particularly in the face of projected increases in both LBP and knee OA prevalence due to an aging population and rising obesity rates, the results of this study are encouraging and provide additional support to previous studies that have demonstrated the cost-effectiveness of this interventions,^21,25^ while using a unique cohort of patients.

Musculoskeletal conditions pose a considerable burden on the healthcare system, with an estimated expenditure of $400 billion annually on healthcare costs, lost productivity, and related expenses in those with chronic back and knee pain.^3^ Moreover, Williams et al suggest that musculoskeletal conditions may increase the risk of chronic disease. In particular, OA appears to increase the risk of developing cardiovascular disease. Therefore, prevention and early treatment of musculoskeletal conditions may play a role in preventing other chronic diseases.^32^ Overall, the results of the current study suggest a saving of roughly $9 million, after accounting for the cost of the intervention, equaling an 81% reduction in the cost of care over 20 months on average. We estimate that the leading reason for the reduction in HCRU was a result of the significant reduction in pain and improvement in function, which met the minimal clinically important difference.^28,33^

These results are consistent with previous publications, including a recent RCT for patients with chronic LBP and multiple studies for patients with knee OA that demonstrate safety and clinical efficacy for those treated with the device.^12,13,22,24,34^ According to Lentz et al, among the modifiable variables associated with increased risk of high expenditure classification are greater pain interference and higher use of prescription medication for pain.^35^ Moreover, Jafarzadeh et al evaluated the relationship between pain reduction and prevention of total knee replacement, which is the main contributor to the high costs associated with knee OA and found a linear correlation between reduction in pain and reduction in knee replacements.^36^

Another potential reason for the reduction in HCRU relates to the biomechanical changes associated with using the device. Those include changes in load distribution and improvement in neuromuscular control.^14,15,17,18,20^ The unique capabilities of the device, which encompass personalized fitting of the device to obtain optimal COP re-positioning and an option to select a pod convexity profile, which determines the level of perturbation to destabilize the joint and stimulate neuromuscular responses, are key capabilities that help address biomechanical anomalies often associated with chronic musculoskeletal conditions.^10,11^ Previous studies have demonstrated that using the device leads to functional realignment (eg, reduction in knee adduction moment) and changes to muscle activation patterns, shifting from co-contraction behavior to a more synchronized contraction pattern between the agonist and antagonist muscles of the lower extremity.

Some limitations should be acknowledged. First, this study used data from a large healthcare service provider rather than directly from the payer. Therefore, we do not have information on other healthcare services patients could potentially receive elsewhere, or if they discontinued insurance. Ideally, direct access to the payer’s claims data set is preferred; however, since the data for this study was obtained by a large service provider for the insurance company, we do not believe a payer’s claim analysis would yield results that are materially different than those presented herein.

Second, the average follow-up duration was roughly 20 months. Although some long-term evidence is available and supports a reduction in rates of knee surgery up to 5 years,^22–26^ future longitudinal studies that look at the entire healthcare expenditure would be beneficial to elucidate long-term benefits and further establish the cost-effectiveness of this intervention. Third, this was a retrospective study with missing data, mainly patient-reported outcome measures of pain, function, and satisfaction. Therefore, the clinical outcomes of this study should be carefully interpreted due to potential bias. That said, the reduction in pain and improvement in function and gait velocity are consistent with previous studies.

## CONCLUSION

In evaluating patient outcomes through both clinical and economic lenses, the current study adds valuable insight to a growing body of literature advocating for innovative, noninvasive treatment strategies. The examined biomechanical interventions led to a significant reduction in pain and improvement in function, which presumably was a driving factor for a meaningful reduction in HCRU and potential cost savings that could alleviate the economic burden on the healthcare system.
